# Head–core temperature discordance during hypothermic circulatory arrest in acute type A aortic dissection

**DOI:** 10.1016/j.xjon.2026.101948

**Published:** 2026-07-02

**Authors:** Hodaka Wakisaka, Noriyuki Takashima, Tomoaki Suzuki

**Affiliations:** a Department of Cardiovascular Surgery, Shiga University of Medical Science, Otsu, Shiga, Japan; b Department of Cardiovascular Surgery, Koto Memorial Hospital, Hiramatsu, Shiga, Japan

**Keywords:** acute type A aortic dissection, hypothermic circulatory arrest, cerebral protection, carotid artery dissection, temperature monitoring

## Abstract

**Objectives:**

Core temperature is routinely used to guide hypothermic circulatory arrest (HCA) for acute type A aortic dissection (ATAAD). However, it remains unclear whether impaired carotid artery flow alters the relationship between cerebral and systemic cooling during HCA. This study aimed to evaluate head–core temperature discordance in patients with carotid artery dissection.

**Methods:**

This retrospective study included 358 consecutive patients who underwent emergency hemiarch replacement for ATAAD under HCA without adjunctive cerebral perfusion between 2012 and 2022. Patients were classified according to carotid artery flow status: no carotid dissection, preserved carotid flow, or compromised carotid flow. A temperature discordance index (Δ*T*) was defined as the difference between bladder (core) and tympanic (head) temperatures at the onset of circulatory arrest.

**Results:**

Tympanic temperature at the onset of circulatory arrest was maintained across all groups (median, 24.5 °C). Postoperative stroke occurred more frequently in patients with carotid artery dissection than in those without carotid involvement (14.8% vs 8.3%; *P* = .04). Patients with compromised carotid flow exhibited a significantly smaller Δ*T* compared with patients without carotid dissection (median, 2.3 °C vs 3.2 °C; *P* < .01). Multivariable logistic regression analysis identified smaller Δ*T* (odds ratio, 0.77) and compromised carotid flow (odds ratio, 2.40) as predictors of postoperative stroke.

**Conclusions:**

During HCA, compromised carotid artery flow is associated with attenuated Δ*T*. A narrowed Δ*T* may reflect impaired cerebral cooling related to compromised carotid perfusion dynamics and was associated with stroke. Monitoring head–core temperature relationships may provide adjunctive intraoperative physiological information beyond core temperature alone.

**Figure:**
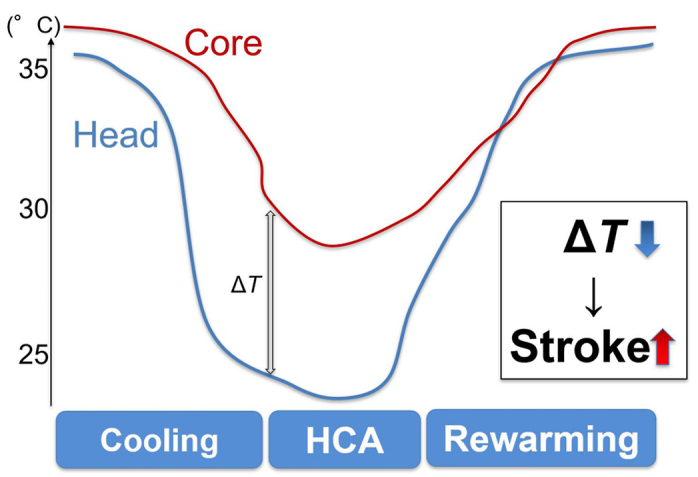
Reduced head–core temperature discordance is associated with postoperative stroke during HCA.

**Central Message**

Narrowed Δ*T* may reflect impaired cerebral cooling and was associated with postoperative stroke.

**Perspective**

Cerebral protection during HCA relies on adequate cooling, but core temperature alone may not reflect cerebral thermal status, particularly in patients with carotid flow impairment. This study highlights head–core temperature discordance as a physiological marker of impaired cerebral cooling and stroke risk, indicating potential to refine intraoperative assessment and guide neuroprotective strategies.

Acute type A aortic dissection (ATAAD) is a life-threatening cardiovascular emergency that requires immediate surgical intervention, with reported postoperative mortality rates ranging from 15% to 30%.^1^ Despite advances in operative techniques and perioperative management, neurologic complications—particularly postoperative stroke—remain a major contributor to early morbidity and mortality after ATAAD repair.^2^^,^^3^

Hypothermic circulatory arrest (HCA) is widely used in aortic surgery to protect the brain and other vital organs. In routine clinical practice, the initiation of circulatory arrest is guided primarily by core temperature measurements, typically obtained from bladder or rectal probes, in accordance with current surgical guidelines.^3^, ^4^, ^5^ However, core temperature may not accurately reflect cerebral temperature, particularly in patients with impaired cerebrovascular flow.^6^ Because neuroprotection during HCA depends on adequate cerebral cooling rather than systemic hypothermia alone, reliance on core temperature alone may be insufficient. Antegrade cerebral perfusion has been increasingly adopted and is associated with improved neurologic outcomes.^7^^,^^8^ However, even with such strategies, the impact of underlying carotid artery pathology on cerebral perfusion and cooling dynamics remains incompletely understood.

Carotid artery involvement is common in ATAAD and can significantly alter cerebral perfusion dynamics. Previous studies have demonstrated that carotid artery dissection—especially when accompanied by true lumen compromise or thrombosis—is associated with an increased risk of neurologic complications.^9^, ^10^, ^11^

We hypothesized that carotid artery dissection, particularly when associated with compromised true lumen flow, would alter cerebral cooling and result in reduced head–core temperature discordance during HCA. To test this hypothesis, we evaluated head–core temperature differences using tympanic and bladder temperature measurements in patients undergoing emergency hemiarch replacement for ATAAD without adjunctive cerebral perfusion. The objectives of this study were to clarify the association between carotid artery dissection patterns and intraoperative head–core temperature discordance and to explore its relationship with postoperative stroke.

## Methods

### Data Availability

The data that support the findings of this study are available from the corresponding author upon reasonable request.

### Ethical Statement

This study was conducted in accordance with the principles of the Declaration of Helsinki and was approved by the institutional review board of Shiga University of Medical Science (approval no. R2021-167; February 15, 2022). The board waived the requirement for written informed consent owing to the retrospective nature of the study. All patient data were anonymized before analysis.

### Study Design, Setting, and Participants

**Figure 1:**
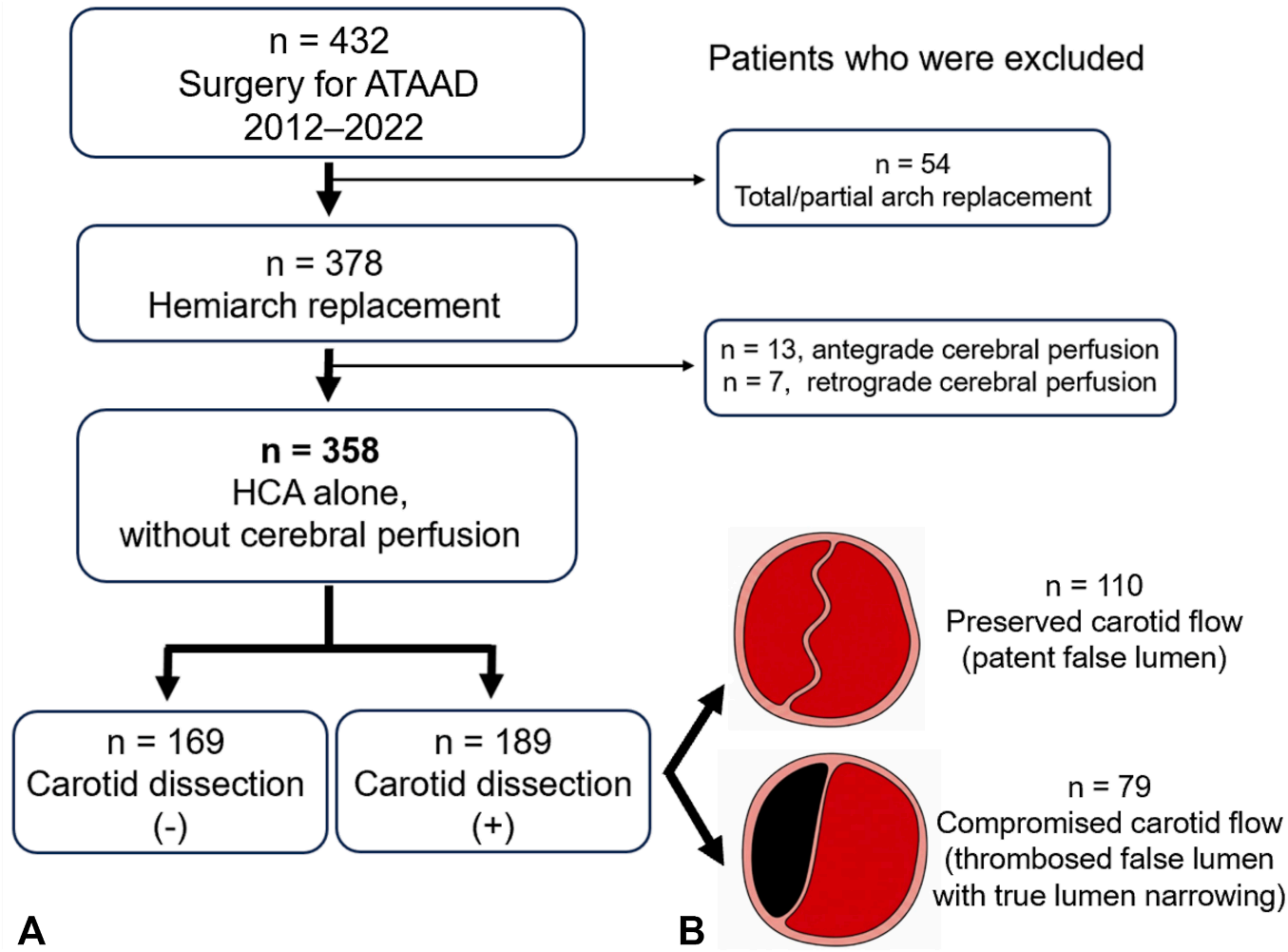
Study flow diagram and classification of carotid artery flow. A, Patient selection flowchart. B, Functional classification of carotid artery dissection based on preoperative computed tomography. *ATAAD*, Acute type A aortic dissection; *HCA*, hypothermic circulatory arrest.

This retrospective, single-center observational study complied with the STROBE (STrengthening the Reporting of OBservational studies in Epidemiology) guidelines for observational retrospective studies. Consecutive patients who underwent emergency surgery for ATAAD at Shiga University of Medical Science between August 2012 and August 2022 were screened for eligibility. Patients who underwent hemiarch replacement under HCA without adjunctive cerebral perfusion were included. Patients who underwent total or partial arch replacement or received antegrade or retrograde cerebral perfusion were excluded (Figure 1). All patients underwent preoperative contrast-enhanced computed tomography (CT) for diagnostic confirmation and anatomical assessment.

Tympanic and bladder temperatures at the onset of circulatory arrest were recorded. Tympanic temperature was used as a surrogate marker of cerebral temperature because of its rapid placement and practicality in emergency settings. Although tympanic temperature may have minor differences compared with measurements that better approximate cerebral temperature, it has been shown to closely reflect trends in cerebral temperature.^6^^,^^12^^,^^13^ Bladder temperature was used to represent systemic core temperature. In patients with anuria attributable to long-term hemodialysis, rectal temperature was used instead of bladder temperature.

Circulatory arrest was generally initiated when the tympanic temperature reached approximately 25 °C, irrespective of core temperature. However, in rare cases in which the core temperature exceeded 30 °C at that time point, cooling was continued until the core temperature decreased to ≤30 °C before circulatory arrest. Head–core temperature discordance (Δ*T*) was defined as the difference between bladder temperature and tympanic temperature at the onset of circulatory arrest (Δ*T* = bladder temperature − tympanic temperature). During cooling, head temperature decreases earlier than systemic core temperature, resulting in a physiological temperature gradient between the 2 compartments. Although Δ*T* was calculated at a single time point, it was considered to reflect the cumulative physiological effects of intraoperative cooling and perfusion.

### Carotid Artery Assessment

Carotid artery involvement was classified on the basis of preoperative CT findings, with particular emphasis on false lumen status and preservation of true lumen flow. Preserved carotid flow was defined as carotid artery dissection with a patent false lumen and maintained antegrade flow within the true lumen. Compromised carotid flow was defined as carotid artery dissection accompanied by false lumen thrombosis, resulting in significant narrowing or occlusion of the true lumen. Patients without carotid artery involvement served as the reference group. In cases of asymmetric carotid artery involvement, patients were classified according to the more severely affected side. Patients with compromised carotid flow were further stratified according to unilateral or bilateral involvement.

### Surgical Technique

The surgical strategy for ATAAD has been described in detail in our previous reports.^14^^,^^15^ In summary, all procedures were performed via a median sternotomy with cardiopulmonary bypass. After the establishment of cardiopulmonary bypass, whole-body cooling was initiated. Circulatory arrest was instituted on the basis of tympanic temperature, with a target of approximately 25 °C. Adjunctive cerebral perfusion was not routinely used and was reserved for selected cases, including those with significant reductions in cerebral oxygen saturation on near-infrared spectroscopy monitoring or when the anticipated circulatory arrest time was expected to exceed 30 minutes because of distal anastomotic complexity. The strategy for temperature management and the criteria for initiating circulatory arrest remained consistent throughout the study period.

### Outcomes

The primary analysis focused on the relationship between carotid flow status and *ΔT* at the onset of circulatory arrest. The secondary analysis evaluated the association between *ΔT* and postoperative stroke. Postoperative stroke was defined as a persistent neurologic deficit with radiologic confirmation of cerebral infarction occurring during the perioperative period.

### Statistical Analysis

Because this was a retrospective observational study including all consecutive eligible patients, no formal a priori sample size calculation was performed. Instead, model parsimony was prioritized, and the number of variables included in the multivariable logistic regression analysis was restricted according to the number of postoperative stroke events to reduce the risk of overfitting and ensure statistical stability.

Continuous distributed variables are presented as medians with interquartile ranges (IQRs). Comparisons were performed using the Student *t* test, Mann-Whitney *U* test, or Kruskal-Wallis test, as appropriate. Categorical variables are expressed as counts and percentages and were compared using the χ^2^ test or Fisher exact test.

All analyses were exploratory and descriptive. To identify factors associated with postoperative stroke, multivariable logistic regression analysis was performed. Variables were selected on the basis of clinical relevance and results of univariable analyses. The final model included age, duration of HCA, presence of compromised carotid flow, and Δ*T*. Statistical analyses were conducted using R software (R Foundation for Statistical Computing).

## Results

A total of 358 patients underwent emergency hemiarch replacement for ATAAD under HCA and were included in the analysis. Carotid artery dissection was identified on preoperative CT in 189 patients (52.8%), whereas 169 patients (47.2%) had no carotid involvement. Baseline characteristics were generally comparable between groups (Table 1). Hypertension was more prevalent among patients with carotid dissection, whereas a history of cerebrovascular disease was more common in patients without carotid involvement.

**Table 1:** Patients’ preoperative characteristics

Characteristics	All (n = 358)	Carotid dissection (+) (n = 189)	Carotid dissection (−) (n = 169)	*P* value
Age, y, mean ± SD	69 ± 12.9	68 ± 12.8	71 ± 12.9	.06
Body mass index, kg/m^2^, mean ± SD	23.3 ± 4.0	23.6 ± 4.0	23.0 ± 4.1	.18
Male	195 (54.5)	106 (56.1)	89 (52.7)	.53
Hypertension	243 (67.9)	137 (72.5)	106 (62.7)	.04
Diabetes mellitus	34 (9.5)	18 (9.5)	16 (9.5)	>.99
Dyslipidemia	101 (28.2)	56 (29.6)	45 (26.6)	.55
Smoking history	143 (39.9)	74 (39.2)	69 (40.8)	.76
Previous cerebrovascular accident	37 (10.3)	14 (7.4)	23 (13.6)	.04
Renal insufficiency	38 (10.6)	19 (10.1)	19 (11.2)	.74
Lung disease	49 (13.7)	24 (12.7)	25 (14.8)	.58
Previous cardiac surgery	11 (3.1)	4 (2.1)	7 (4.1)	.29
Malperfusion				
Coronary	11 (3.1)	6 (3.2)	5 (3.0)	.91
Visceral, renal	38 (10.6)	21 (11.1)	17 (10.1)	.77
Extremities	30 (8.4)	19 (10.1)	11 (6.5)	.22
Aortic valve insufficiency	44 (12.3)	27 (14.3)	17 (10.1)	.23
Neurologic deficit	67 (18.7)	38 (20.1)	29 (17.2)	.49
Hemodynamic instability	51 (14.2)	21 (11.1)	30 (17.8)	.07
Cardiopulmonary resuscitation	22 (6.1)	9 (4.8)	13 (7.7)	.24

Values are n (%) unless otherwise indicated.

Cardiopulmonary bypass duration, aortic crossclamp time, and circulatory arrest time did not differ significantly between groups (Table 2). Tympanic temperature at the onset of circulatory arrest was consistently controlled according to the institutional cooling protocol and showed no significant intergroup differences. In contrast, bladder temperature exhibited substantial interindividual variability, resulting in a wide distribution of Δ*T*.

**Table 2:** Operative data and outcomes

Variables	All (n = 358)	Carotid dissection (+) (n = 189)	Carotid dissection (−) (n = 169)	*P* value
Cannulation site				
Ascending artery	22 (6.1)	12 (6.3)	10 (5.9)	.88
Axillary artery	56 (15.6)	27 (14.3)	29 (17.2)	.46
Femoral artery	272 (76.0)	144 (76.2)	128 (75.7)	.92
Axillary + femoral artery	8 (2.2)	6 (3.2)	2 (1.2)	.20
Duration of procedure, min, median [IQR]				
Cardiopulmonary bypass time	97 [88-112]	99 [89-112]	97 [87-112]	.51
Crossclamp time	46 [39-55]	46 [39-56]	46 [39-54]	.79
Circulatory arrest time	20 [17-23]	20 [17-23]	19 [17-23]	.61
Nadir temperature, °C, median [IQR]				
Tympanic	24.5 [23.2-25.0]	24.7 [23.8-25.0]	24.3 [23.2-25.0]	.32
Bladder or rectal	27.9 [26.2-28.8]	27.0 [25.3-28.2]	27.4 [26.2-28.6]	.04
Δ*T*, °C	3.9 [2.0-4.9]	2.7 [0.7-4.1]	3.2 [1.9-5.0]	<.01
Concomitant operation				
Aortic root replacement	5 (1.4)	3 (1.6)	2 (1.2)	.76
Aortic valve repair or replacement	23 (6.4)	11 (5.8)	12 (7.1)	.63
Coronary bypass	26 (7.3)	17 (9.0)	9 (5.3)	.18
Lower-limb bypass	16 (4.5)	12 (6.3)	4 (2.4)	.05
30-d mortality	23 (6.4)	13 (6.9)	10 (5.9)	.71
Stroke	42 (11.7)	28 (14.8)	14 (8.3)	.04
Spinal ischemia	8 (2.2)	5 (2.6)	3 (1.8)	.62
Newly hemodialysis	24 (6.7)	14 (7.4)	10 (5.9)	.57
Reoperation for bleeding	52 (14.5)	30 (15.9)	22 (13.0)	.43
Prolonged ventilation	28 (7.8)	12 (6.3)	16 (9.5)	.27
GI complication	12 (3.4)	6 (3.2)	6 (3.6)	.83
Postoperative IABP or PCPS	10 (2.8)	4 (2.1)	6 (3.6)	.40

Values are n (%) unless otherwise indicated. *IQR*, Interquartile range; *ΔT*, head–core temperature discordance; *GI*, gastrointestinal; *IABP*, intra-aortic balloon pump; *PCPS*, percutaneous cardiopulmonary support.

**Figure 2:**
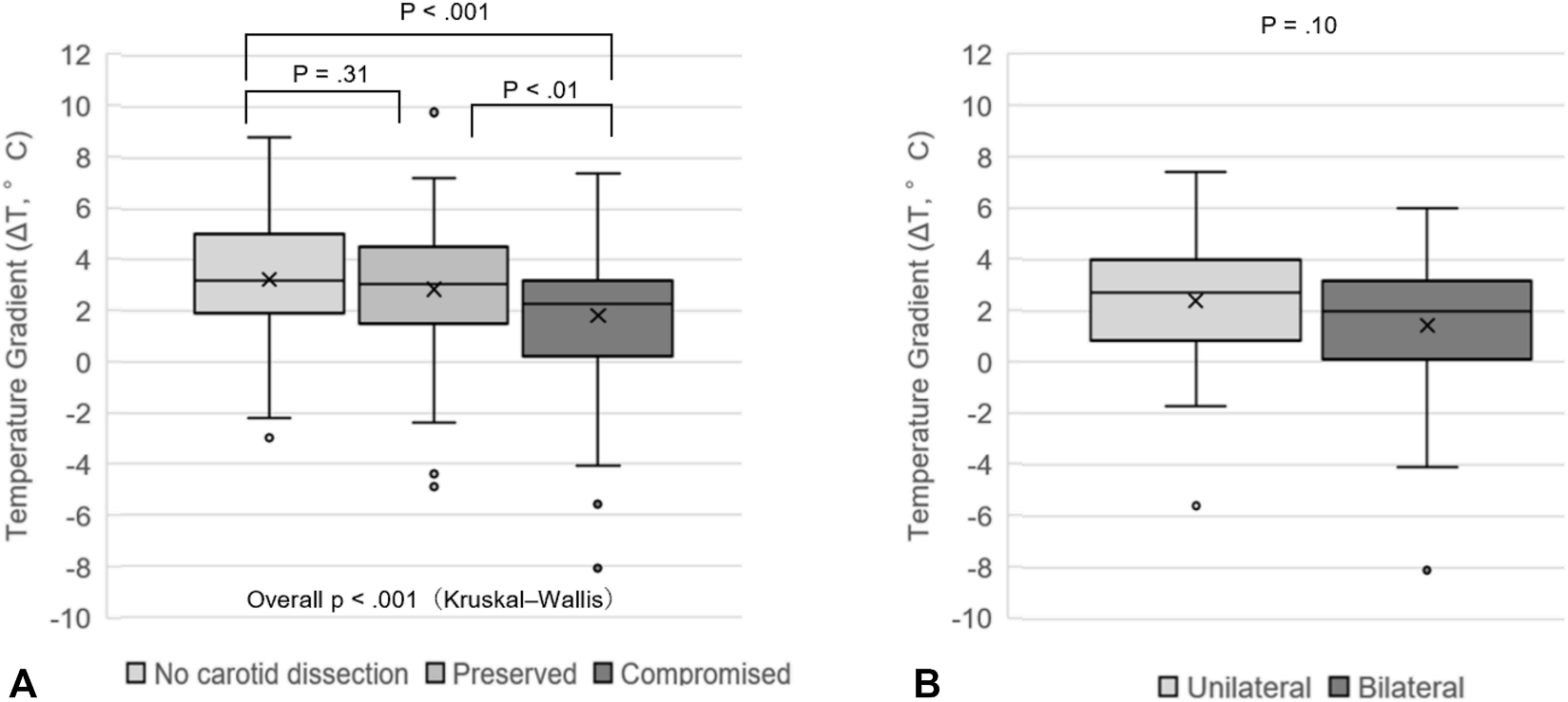
Head–core temperature discordance (Δ*T*) stratified by carotid flow status. A, Comparison of Δ*T* among patients without carotid dissection, with preserved carotid flow, and with compromised carotid flow. B, Comparison of Δ*T* between unilateral and bilateral carotid artery involvement in patients with compromised carotid flow. *Boxes* represent interquartile ranges with medians; *crosses* indicate means.

Patients were further stratified into 3 groups on the basis of carotid artery status: no carotid dissection, carotid dissection with preserved flow, and carotid dissection with compromised flow. The distribution of Δ*T* differed significantly among these groups (Figure 2, *A*). Δ*T* was similar between patients without carotid dissection and those with preserved carotid flow (median [IQR], 3.2 [1.9-5.0] vs 3.1 [1.5-4.5]; *P* = .31). In contrast, patients with compromised carotid flow demonstrated a significantly narrower Δ*T* (median [IQR], 2.3 [0.2-3.2]) compared with the preserved-flow group (*P* = .008) and patients without carotid dissection (*P* < .01). Among patients with compromised carotid flow, Δ*T* tended to be smaller in those with bilateral involvement than in those with unilateral involvement; however, this difference did not reach statistical significance (*P* = .10; Figure 2, *B*).

**Figure 3:**
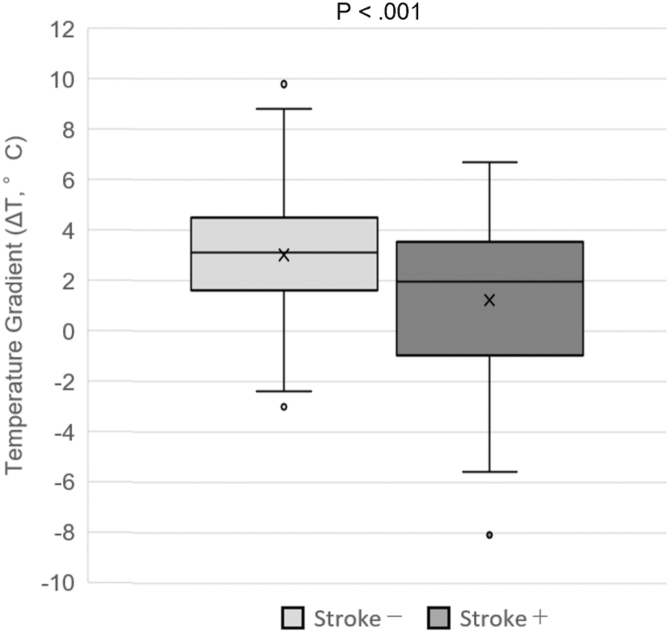
Relationship between head–core temperature discordance (Δ*T*) and postoperative stroke. Comparison of Δ*T* between patients with and without postoperative stroke. *Boxes* represent interquartile ranges with medians; *crosses* indicate means.

Postoperative stroke occurred in 42 patients (11.7%). Given the observed association between carotid flow status and Δ*T*, the relationship between temperature discordance and neurologic outcomes was further examined. Patients who developed postoperative stroke exhibited significantly lower Δ*T* values than those without stroke (median [IQR], 0.35 [−1.65 to 2.1] vs 3.1 [1.6-4.5]; *P* < .01, Figure 3). Multivariable logistic regression analysis identified a smaller Δ*T* (odds ratio, 0.77; 95% CI, 0.68-0.88; *P* < .01) and the presence of compromised carotid flow (odds ratio, 2.40; 95% CI, 1.19-4.84; *P* = .015) as factors associated with postoperative stroke (Table 3).

**Table 3:** Multivariable logistic regression analysis for factors associated with postoperative stroke

Variable	Odds ratio	95% CI	*P* value
Δ*T*, °C	0.77	0.68-0.88	<.001
Compromised carotid flow	2.40	1.19-4.84	.015
Age, y	0.98	0.95-1.00	.07
HCA duration, min	1.02	0.95-1.09	.67

*ΔT*, Head–core temperature discordance; *HCA*, hypothermic circulatory arrest.

## Discussion

This study demonstrates that carotid artery pathology significantly influences cooling dynamics during HCA in patients undergoing emergency hemiarch replacement for ATAAD. The principal findings indicate that functional impairment of carotid blood flow is associated with attenuation of the Δ*T* at the onset of circulatory arrest. Importantly, a narrowed Δ*T* was significantly associated with postoperative stroke in multivariable analysis. These observations suggest that intraoperative temperature dynamics may provide a clinically relevant physiological signal of cerebral risk. Despite being derived from the same institutional database as our previous report on HCA duration, the present study addresses a distinct physiological question regarding the influence of carotid artery flow on Δ*T*.^14^ Previous work focused on arrest duration, whereas Δ*T* offers complementary physiological information beyond duration-based assessments. Although a physiological correlation between carotid flow compromise and narrowed Δ*T* is anticipated, both factors remained associated with postoperative stroke after multivariable adjustment. This suggests that Δ*T* provides incremental prognostic information; nevertheless, it should be interpreted primarily as a downstream physiological manifestation of carotid flow impairment rather than an independent mechanistic driver.

Hypothermia remains a cornerstone of cerebral protection during ATAAD repair when circulatory arrest is required. In routine clinical practice, both head and core temperatures are monitored, with current guidelines emphasizing core temperature as the primary indicator of systemic hypothermia.^2^ However, effective neuroprotection depends on adequate cerebral cooling rather than systemic hypothermia alone. Previous experimental and clinical studies have shown that core temperature may not reliably reflect cerebral temperature, particularly in the presence of altered cerebral perfusion.^6^^,^^16^ Our findings extend this evidence by demonstrating that carotid flow status plays a critical role in determining the relationship between head and core temperatures during cooling.

The observed narrowing of Δ*T* in patients with compromised carotid flow can be explained by differential cooling efficiency between the cerebral and systemic compartments. In clinical practice, circulatory arrest is initiated only after the tympanic temperature reaches the target level (approximately 25 °C). In the presence of carotid flow compromise, cerebral cooling is delayed due to restricted arterial inflow. Consequently, achieving the target tympanic temperature may require prolonged or intensified cooling phase during cardiopulmonary bypass. During this extended cooling period, the systemic compartment—represented by bladder temperature and supported by preserved perfusion—continues to cool efficiently. This results in relative systemic overcooling, thereby reducing Δ*T* at the time of arrest. Thus, paradoxically, a narrowed Δ*T* reflects impaired cerebral heat exchange attributable to compromised carotid perfusion.

Although preoperative factors such as the National Institutes of Health Stroke Scale score and ischemic duration are well-established determinants of neurologic outcomes,^17^, ^18^, ^19^ vascular conditions in ATAAD are inherently dynamic and may evolve between diagnostic imaging and the initiation of HCA. As a result, preoperative imaging may not accurately represent real-time cerebral perfusion status during surgery. In this context, ΔT may serve as an adjunctive intraoperative physiological signal, reflecting impaired cerebral cooling associated with the patient's current carotid flow status. Rather than representing a simple temperature difference, a reduced Δ*T* may indicate that effective cerebral cooling is being compromised by ongoing perfusion abnormalities. Near-infrared spectroscopy is widely used for cerebral monitoring during aortic surgery.^20^^,^^21^ Other intraoperative monitoring modalities have also been explored for dynamic assessment of cerebral perfusion during ATAAD repair. Augoustides and colleagues^22^ have suggested that adjunctive intraoperative vascular imaging modalities, such as carotid ultrasonography and transpharyngeal imaging, may provide additional real-time information regarding cerebral and brachiocephalic perfusion during ATAAD repair. In this context, *ΔT* may be another physiological parameter reflecting dynamic cerebral cooling and perfusion status during HCA, potentially complementing existing intraoperative monitoring strategies.

These physiological insights have important clinical implications. Although a persistently narrowed Δ*T* was strongly associated with postoperative stroke, it should be emphasized that Δ*T* itself is not a causal mechanism of injury. Rather, it functions as a real-time physiological indicator of compromised carotid perfusion. In ATAAD, reliance on anatomical assessment alone may underestimate neurologic risk. The absence of expected temperature divergence may serve as a surrogate marker of hemodynamically significant flow limitation, thereby alerting the surgical team to impending cerebral ischemia before irreversible injury occurs. Specifically, a narrowed Δ*T* may alert surgeons to the possibility of inadequate cerebral cooling and may prompt further intraoperative evaluation of cerebral perfusion adequacy. In such circumstances, prolonging the cooling phase, adding adjunctive cerebral perfusion, or considering intraoperative carotid flow assessment may warrant consideration.

Although bilateral carotid involvement was associated with greater variability and more extreme temperature discordance, laterality alone did not independently determine Δ*T*. Instead, functional carotid flow status—preserved versus compromised—emerged as the primary determinant of cooling behavior. This finding underscores the importance of functional perfusion assessment over anatomical classification when evaluating cerebral protection during HCA, consistent with previous observations emphasizing physiological flow status rather than morphologic extent.

A notable strength of this study is the relatively uniform surgical strategy applied to most patients. The majority underwent hemiarch replacement under HCA without routine adjunctive cerebral perfusion. Although this approach is not intended to discourage the use of selective cerebral perfusion, it allowed for the assessment of intrinsic cooling behavior and perfusion-dependent temperature dynamics that may be obscured in cohorts managed with more complex neuroprotective strategies. This consistency enabled a focused physiological evaluation of head–core temperature relationships during cooling.

### Study Limitations

First, this cohort represents a selected operative strategy without routine adjunctive cerebral perfusion. Although this may limit direct generalizability to contemporary arch surgery, it allowed for clearer characterization of intrinsic cerebral–systemic cooling dynamics under standardized conditions. Therefore, these findings should be interpreted as physiological and hypothesis-generating rather than immediately practice-changing. Second, patients with the most severe cerebral malperfusion requiring adjunctive cerebral perfusion or total arch replacement may have been underrepresented, potentially limiting assessment across the full spectrum of carotid flow compromise. Third, carotid artery involvement was determined using preoperative imaging, despite the dynamic nature of ATAAD pathology, which may evolve between imaging and surgery. Fourth, the impact of arterial cannulation site on cooling dynamics was not determined and may have affected temperature distribution. Finally, this retrospective, single-center design limits generalizability.

## Conclusions

Compromised carotid artery flow was associated with narrowing of the Δ*T* during HCA in ATAAD, and a smaller Δ*T* was associated with postoperative stroke after multivariable adjustment. Accordingly, reliance on core temperature monitoring in isolation may be insufficient.

## Conflict of Interest Statement

The authors reported no conflicts of interest.

The *Journal* policy requires editors and reviewers to disclose conflicts of interest and to decline handling or reviewing manuscripts for which they may have a conflict of interest. The editors and reviewers of this article have no conflicts of interest.
